# Supplementary figures and images for: Stage- and tissue-specific gene editing using 4-OHT–inducible Cas9 in whole organism
Source: J Cell Biol. 2026 Jan 2;225(4):e202412216. doi: 10.1083/jcb.202412216 (PMC12758452; doi:10.1083/jcb.202412216)

Figure 1 -1

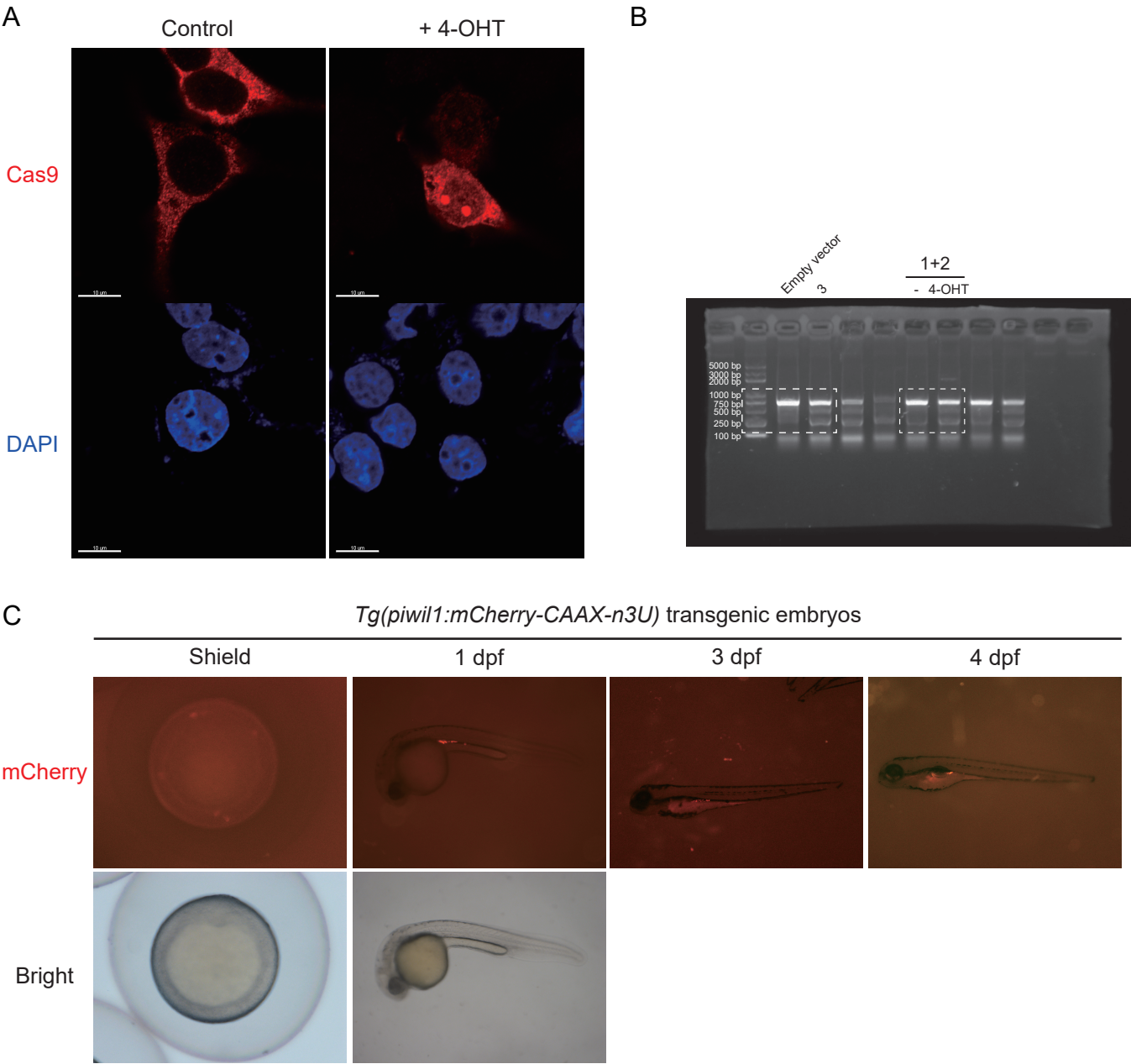

Figure 1 -2

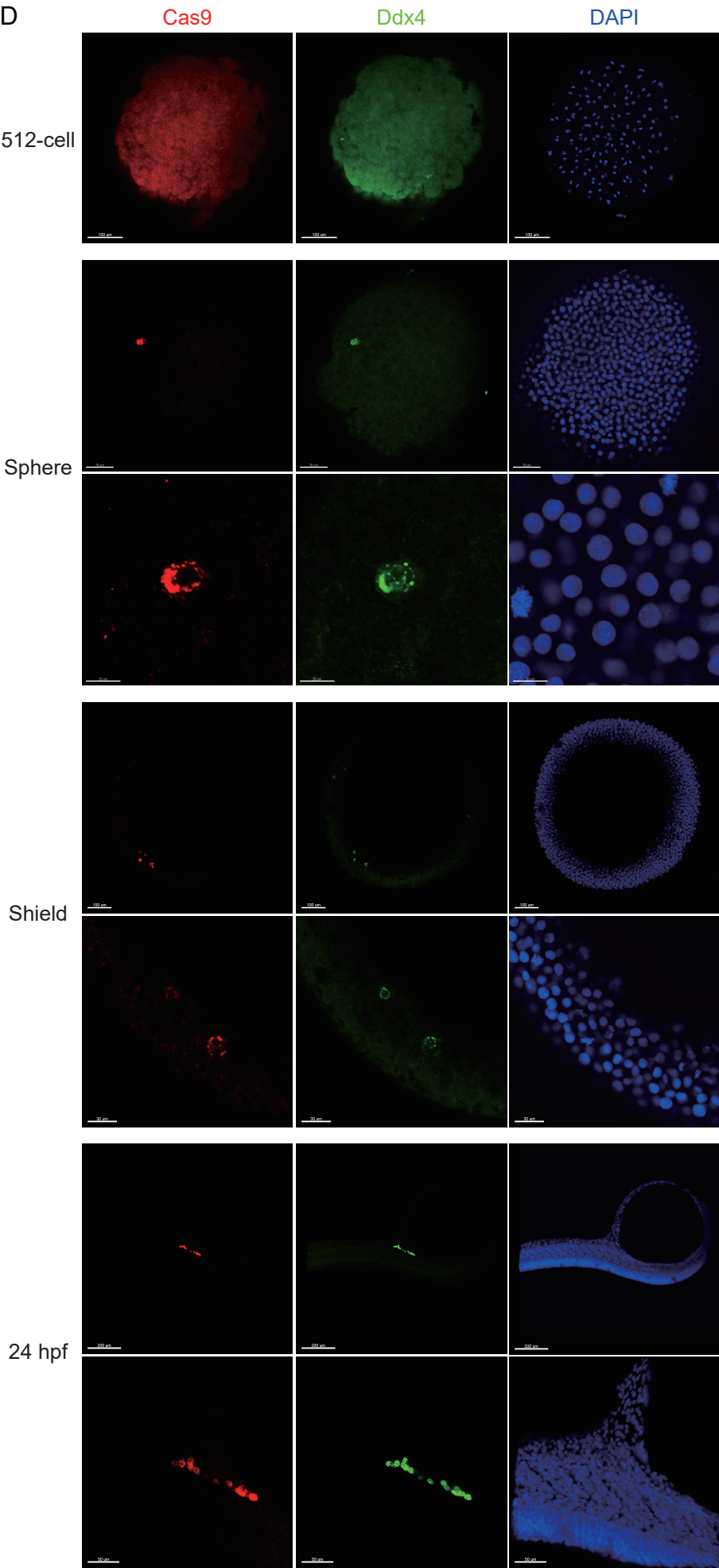

Supplement: SourceData F1 — is the source file for Fig. 1. [file jcb_202412216_sourcedataf1.pdf]

Figure 2

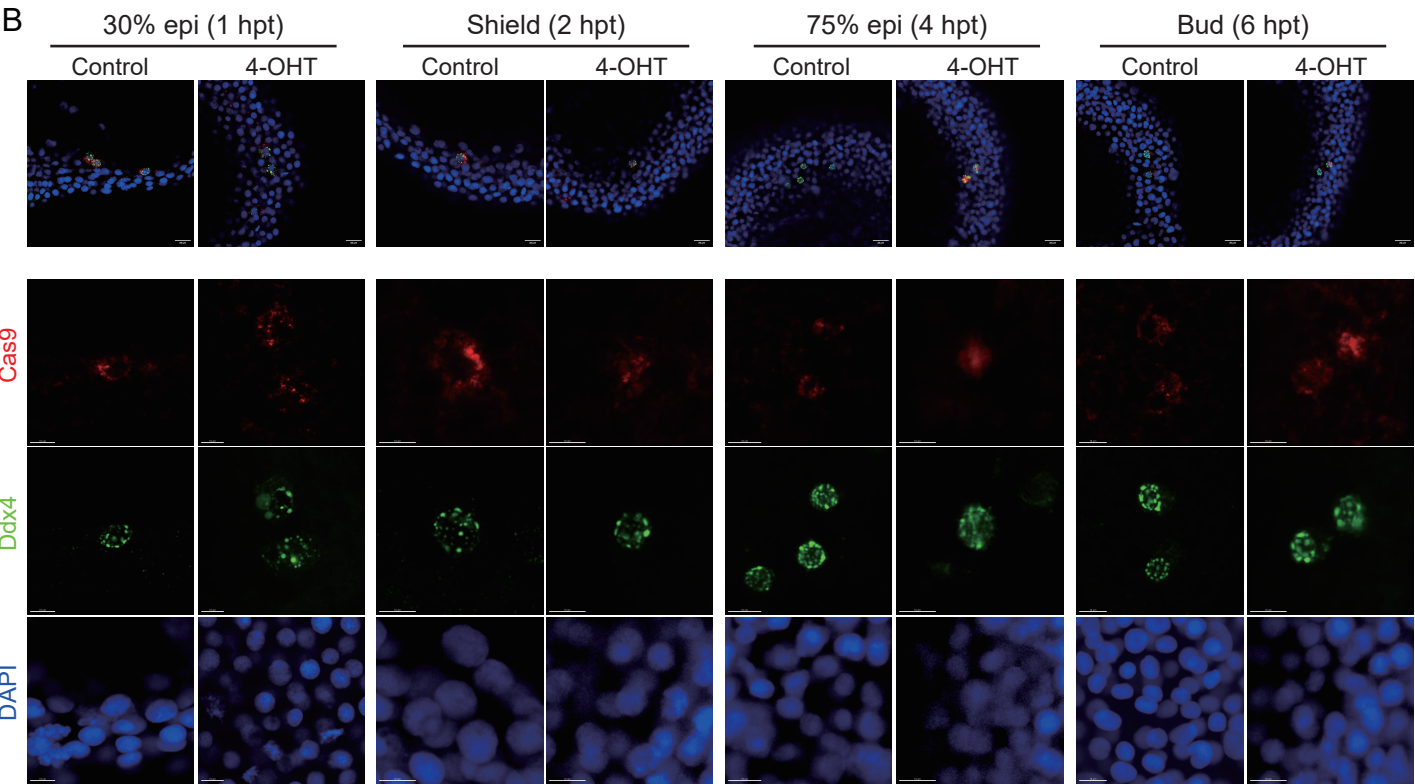

Supplement: SourceData F2 — is the source file for Fig. 2. [file jcb_202412216_sourcedataf2.pdf]

Figure 3

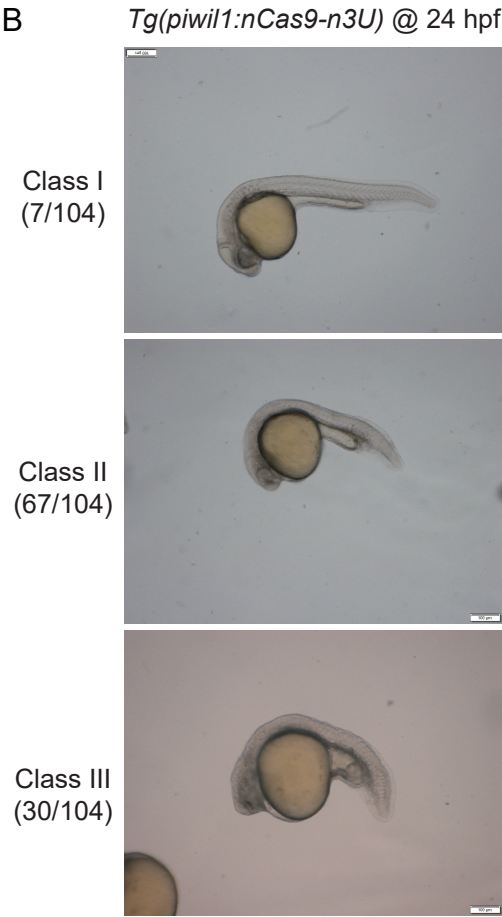

Supplement: SourceData F3 — is the source file for Fig. 3. [file jcb_202412216_sourcedataf3.pdf]

Figure 5

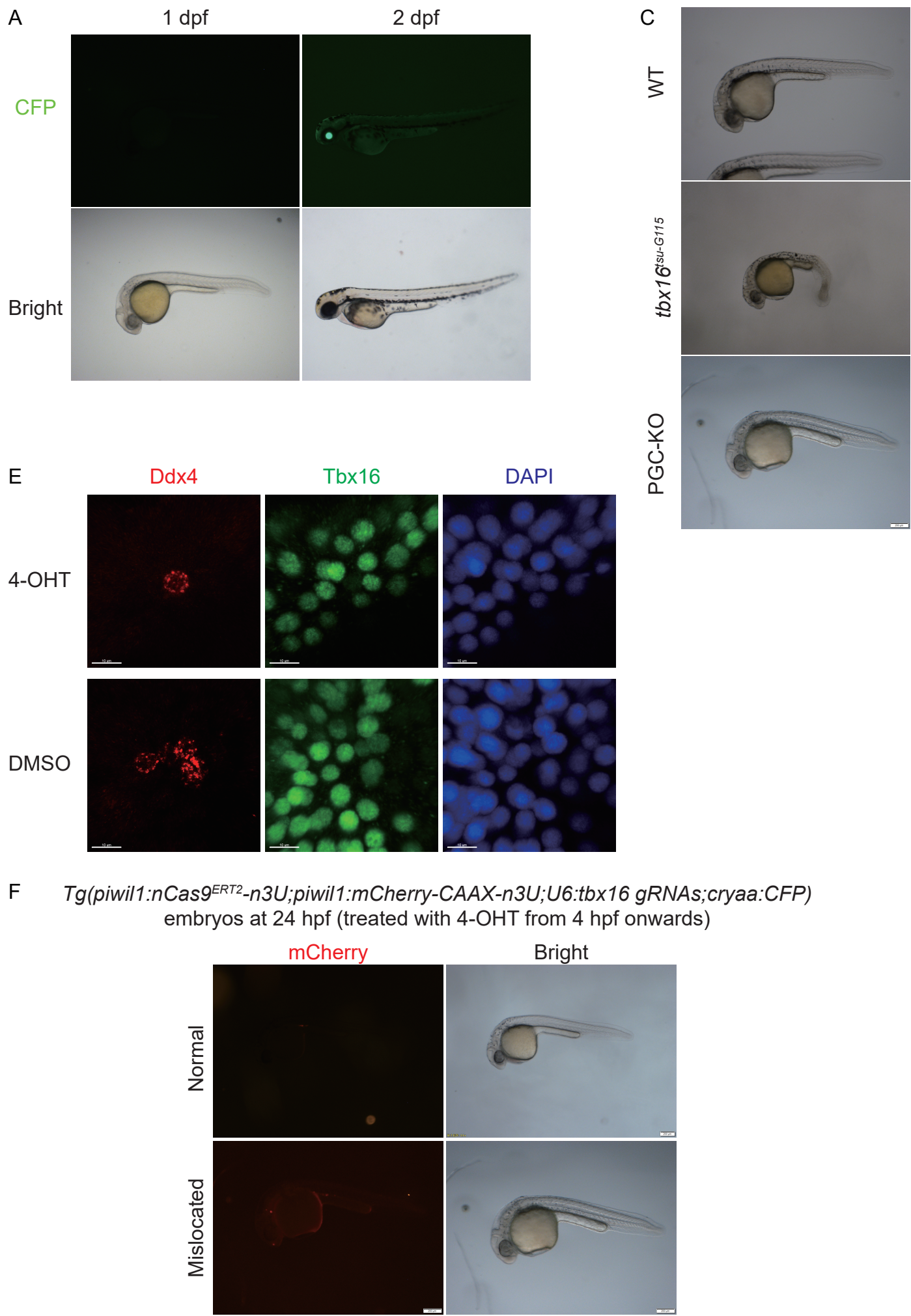

Supplement: SourceData F5 — is the source file for Fig. 5. [file jcb_202412216_sourcedataf5.pdf]

Figure 6

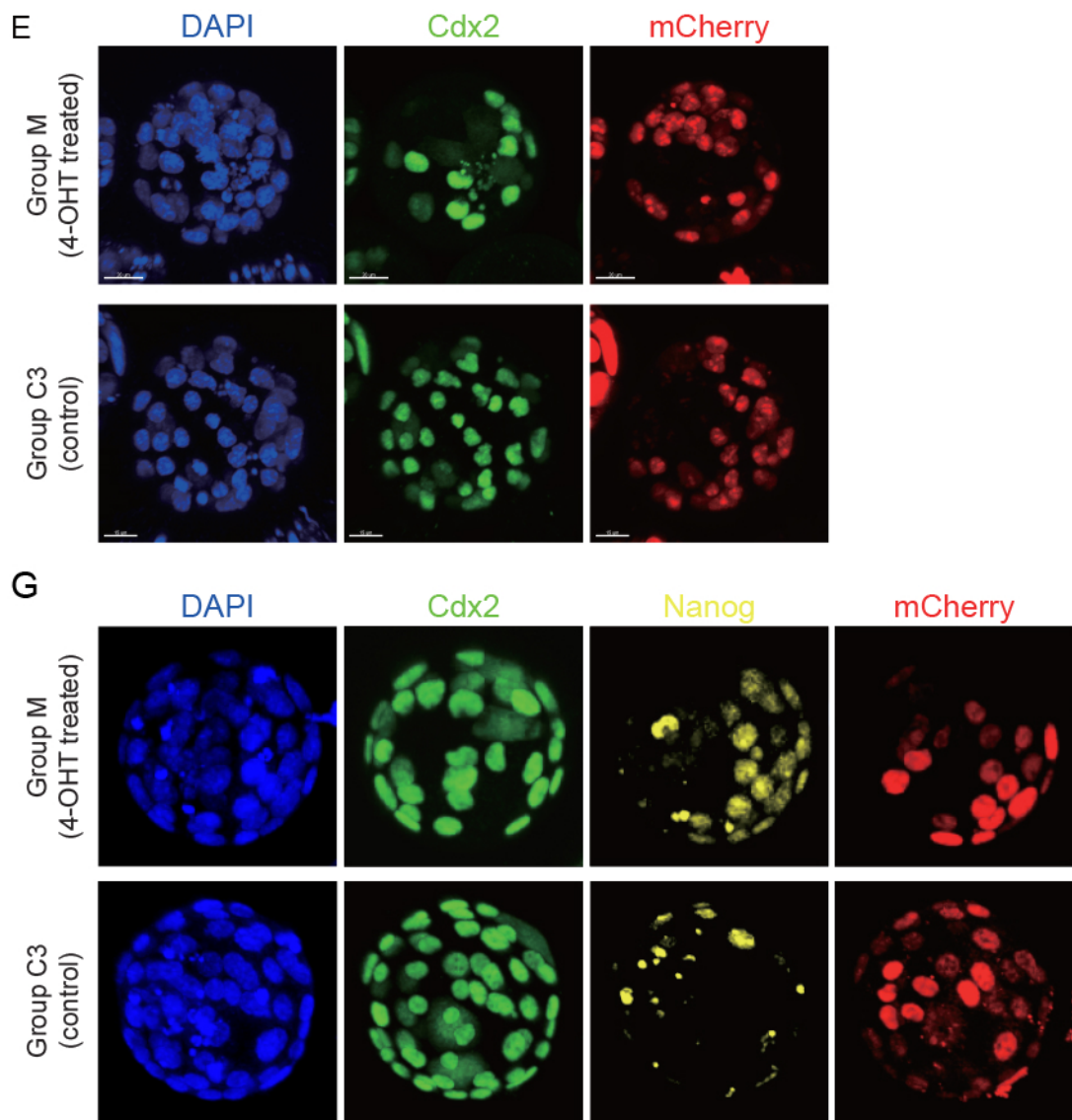

Supplement: SourceData F6 — is the source file for Fig. 6. [file jcb_202412216_sourcedataf6.pdf]

Figure S1

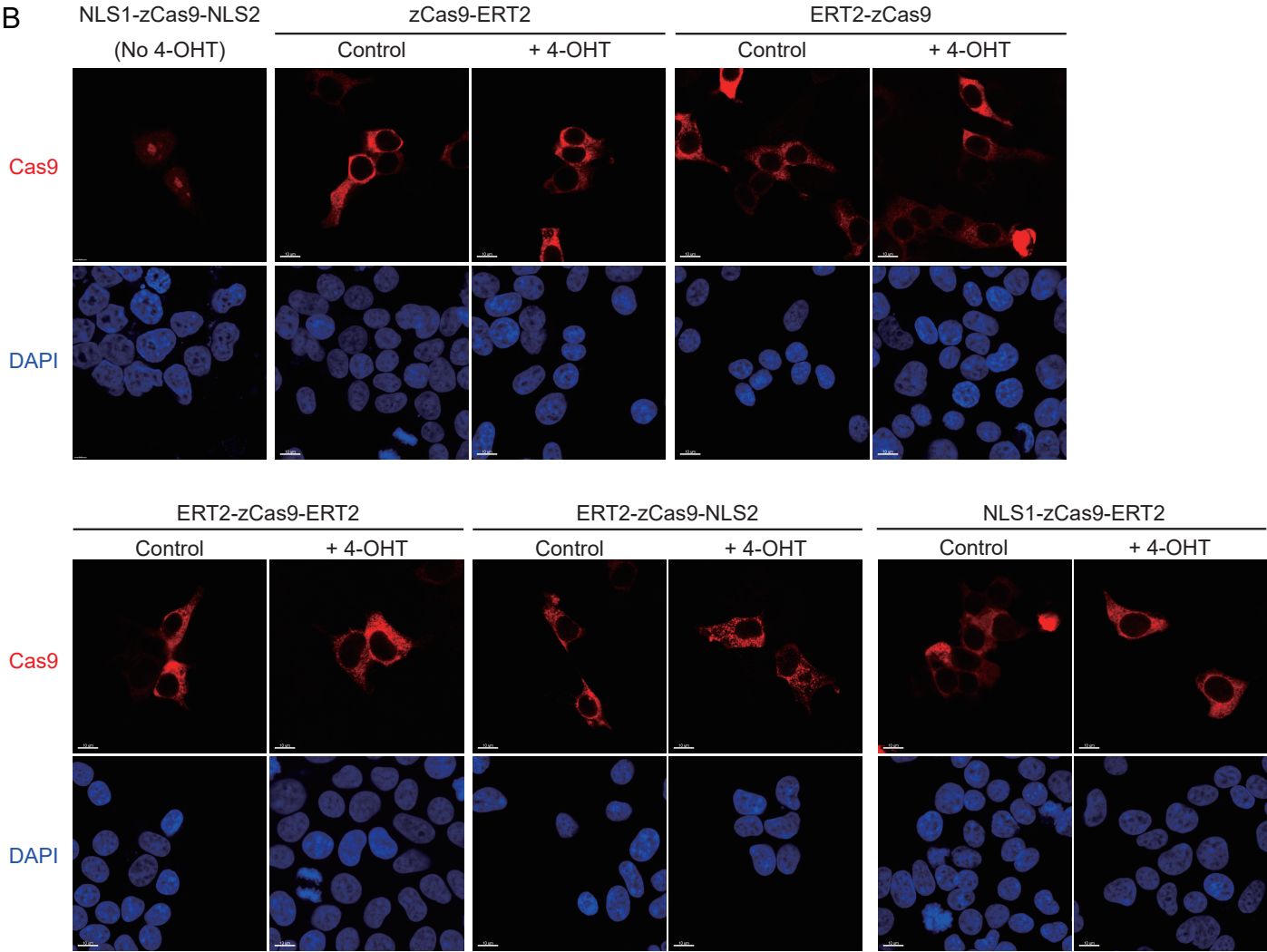

Supplement: SourceData FS1 — is the source file for Fig. S1. [file jcb_202412216_sourcedatafs1.pdf]

Figure S2 -1

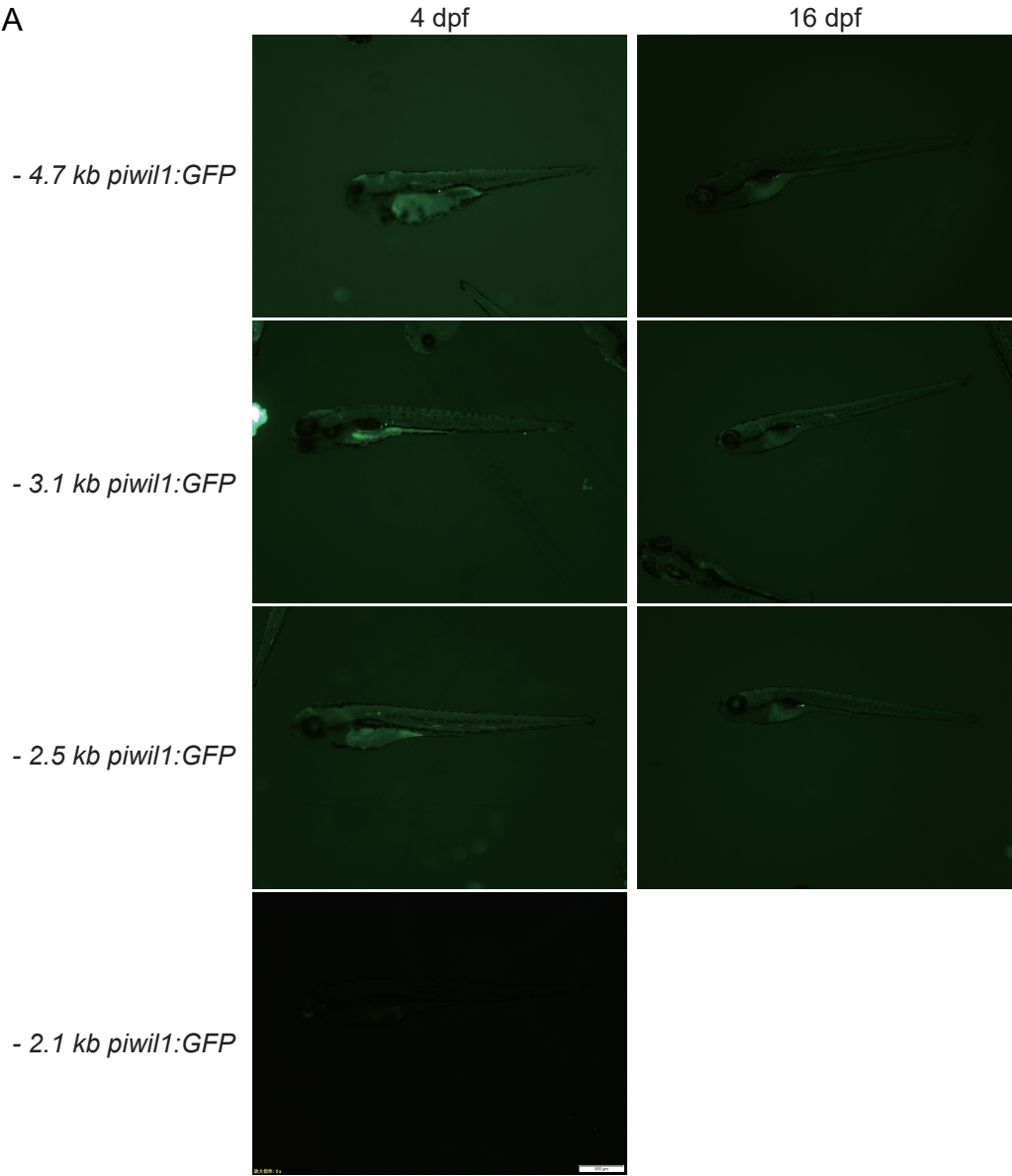

Figure S2 -2

B *Tg(piwil1:mCherry-CAAX-n3U)* transgenic embryos

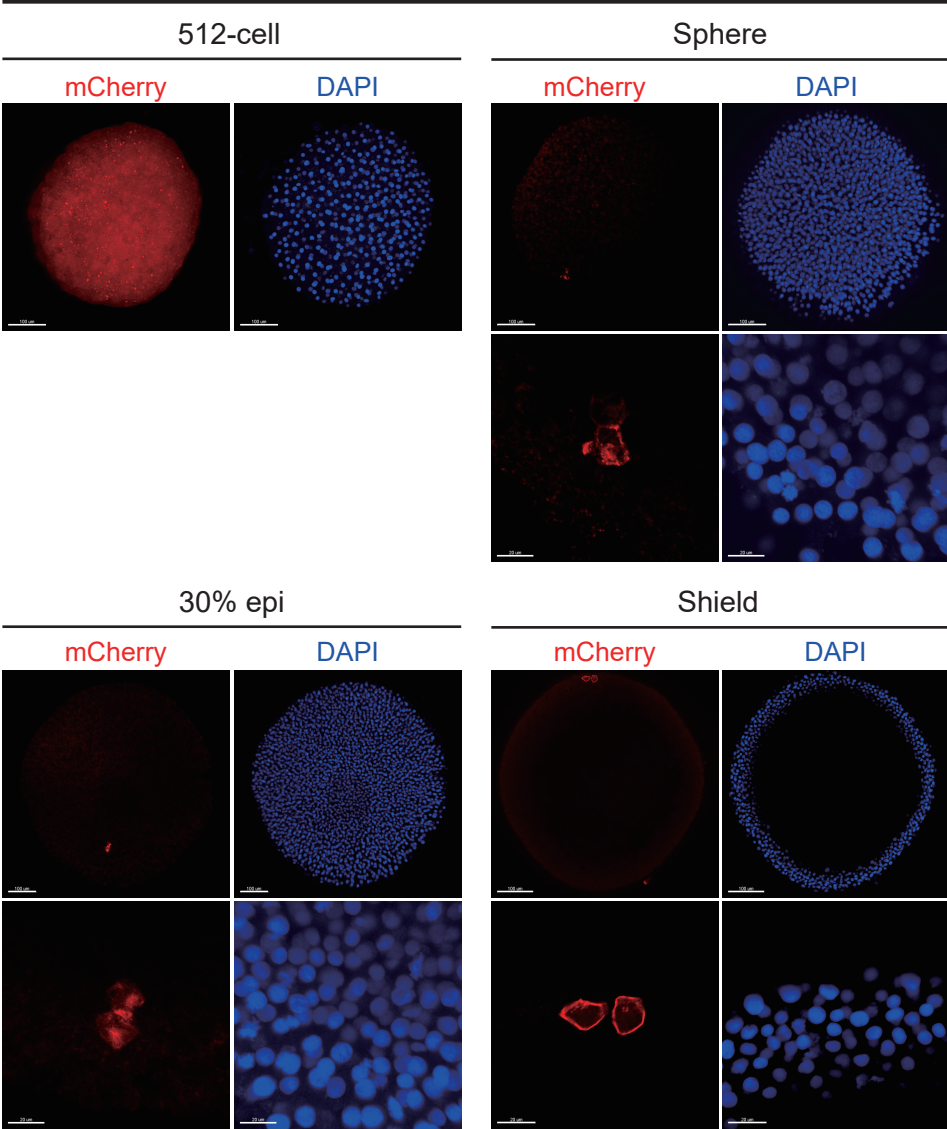

Supplement: SourceData FS2 — is the source file for Fig. S2. [file jcb_202412216_sourcedatafs2.pdf]

Figure S4

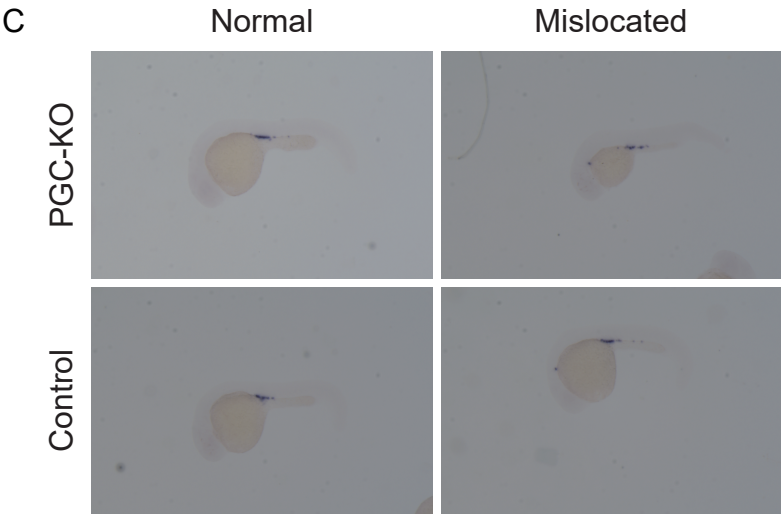

Supplement: SourceData FS4 — is the source file for Fig. S4. [file jcb_202412216_sourcedatafs4.pdf]

Figure S5

C

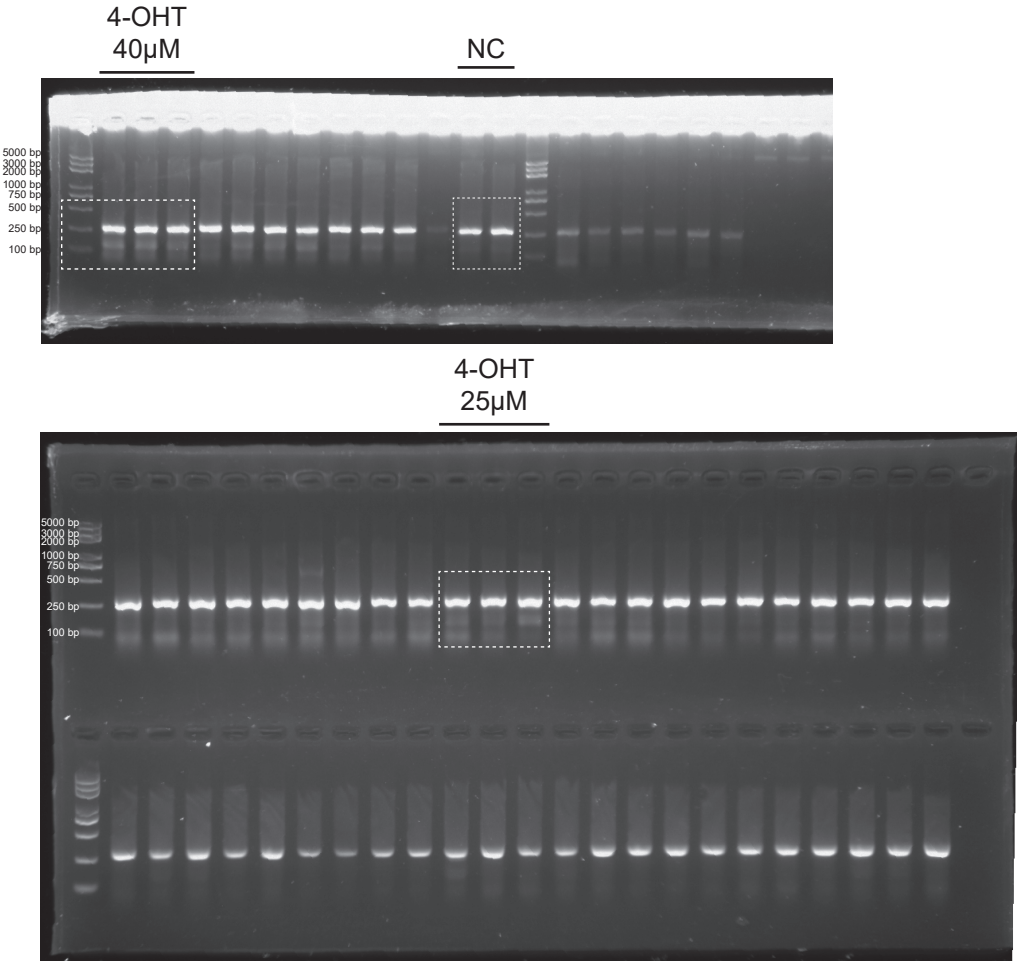

D

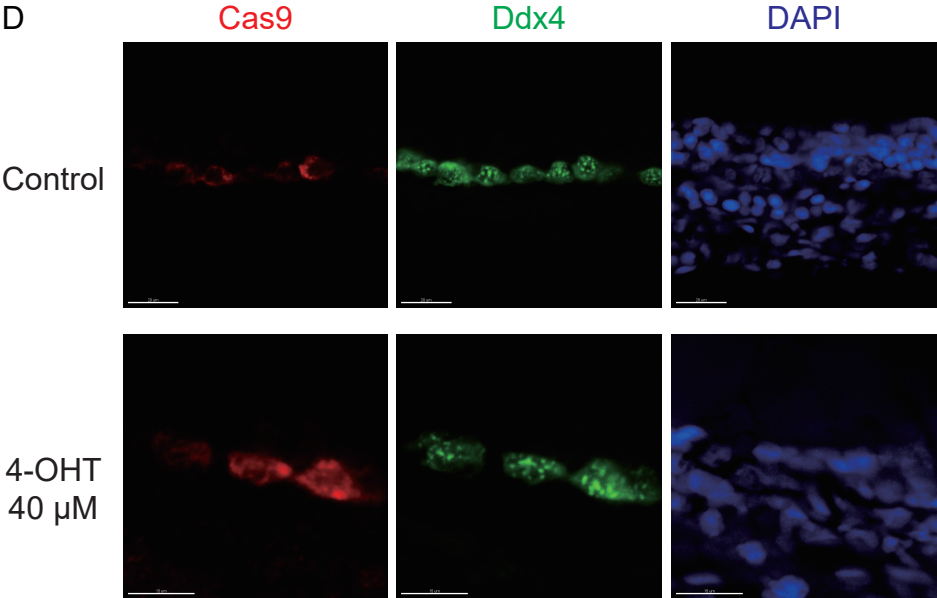

Supplement: SourceData FS5 — is the source file for Fig. S5. [file jcb_202412216_sourcedatafs5.pdf]
