# Supplementary material for: Stage- and tissue-specific gene editing using 4-OHT–inducible Cas9 in whole organism
Source: J Cell Biol. 2026 Jan 2;225(4):e202412216. doi: 10.1083/jcb.202412216 (PMC12758452; doi:10.1083/jcb.202412216)
Supplement: SourceData F4 — is the source file for Fig. 4. [file jcb_202412216_sourcedataf4.pdf]

Figure 4

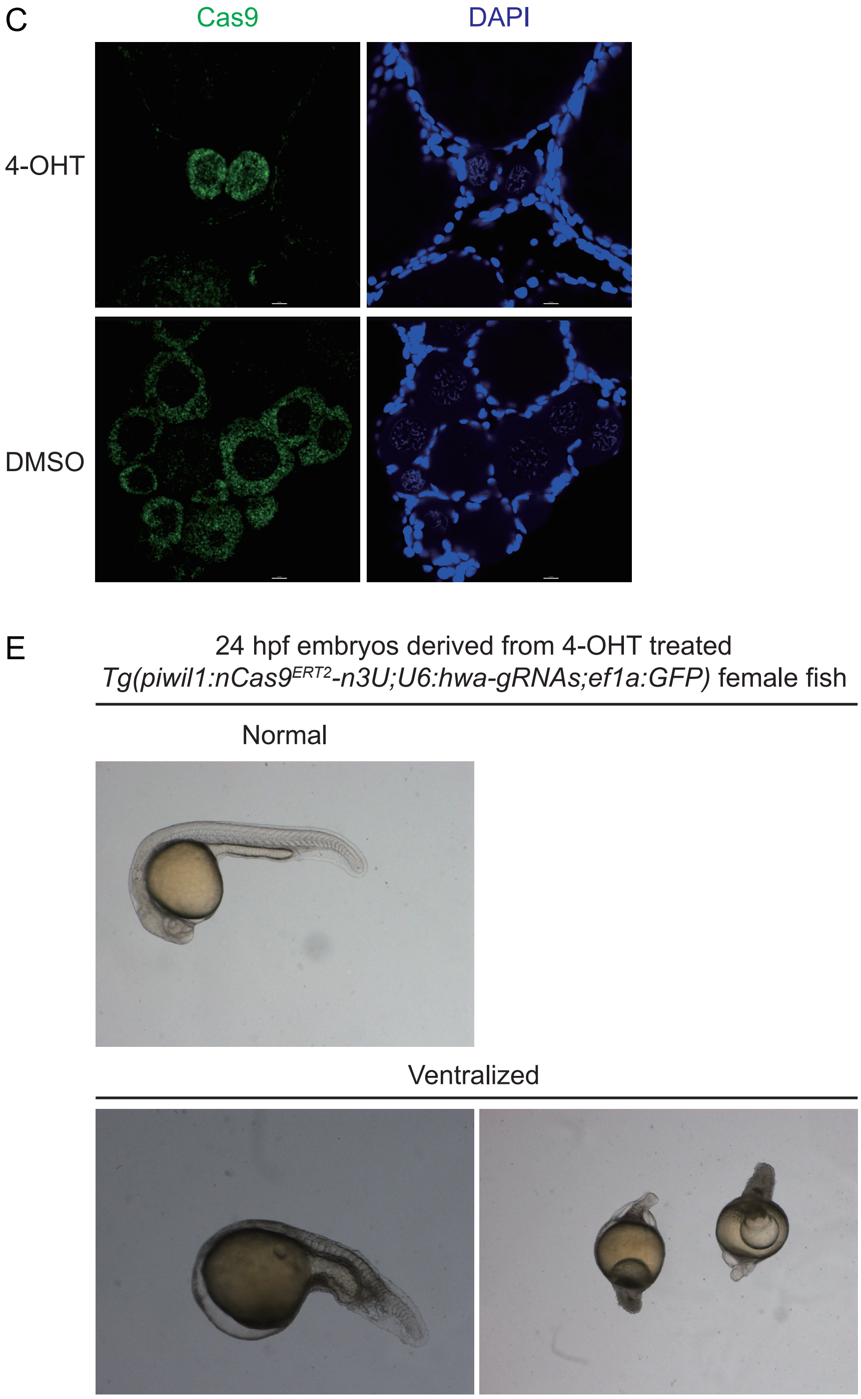
C
Cas9
DAPI
4-OHT
DMSO
E
24 hpf embryos derived from 4-OHT treated
Tg(piwil1:nCas9^{ERT2}-n3U;U6:hwa-gRNAs;ef1a:GFP) female fish
Normal
Ventralized
